# Peptide vaccines designed with the aid of immunoinformatic against Caseous Lymphadenitis promotes humoral and cellular response induction in mice

**DOI:** 10.1371/journal.pone.0256864

**Published:** 2021-11-29

**Authors:** Daniela Droppa-Almeida, Glenda Amaral da Silva, Lívia Maria do Amorim Costa Gaspar, Beatriz Benny Sungaila Pereyra, Roberto José Meyer Nascimento, Sibele Borsuk, Elton Franceschi, Francine Ferreira Padilha

**Affiliations:** 1 Center for Studies on Colloidal Systems (NUESC)/Institute of Technology and Research (ITP), Tiradentes University (UNIT), Aracaju, Brazil; 2 Institute of Health Science, Department of Immunology, Federal University of Bahia (UFBA), Salvador, Brazil; 3 Technological Development Center, Biotechnology, Federal University of Pelotas (UFPel), Campus Universitário, Pelotas, Brazil; Xinyang Normal University, CHINA

## Abstract

Caseous Lymphadenitis (CLA) is a chronic disease that affects also small ruminants. CLA is caused by *Corynebacterium pseudotuberculosis* and is responsible for high economic losses due to the formation of superficial and visceral granulomas, the latter is considered as asymptomatic CLA causing high levels of dissemination. Several vaccination strategies, in which the use of synthetic peptides stands out. Thus, this work aimed to evaluate the protective potential of peptide vaccines designed to determine the immunodominant epitopes of CP40 against CLA in mice. The animals were divided into eight groups separated in controls (G1—PBS, G2—Saponin and G9—rCP40) and experimental (G3—pep1, G4- pep2, G5-pep3, G6-pep4, G7-pep5 and G8-pep6), these were vaccinated on days 0 and 15 by a subcutaneous route. 60 days after the first immunization, all animals were challenged with *C*. *pseudotuberculosis*. On days 0, 15, 60, and 120 after the first immunization, blood samples were taken to measure immunoglobulins. On the same day of the challenge, the splenocytes were isolated and assayed for the production of IL-2, IL-4, IL-6, IFN-γ, TNF-α, IL-17, and IL-10. After vaccinations, the animals were challenged and all of them were affected by the disease which led to their death. The G6 and G8 groups provided 10% protection and the G7 provided 20%. The G3 and G4 groups provided 30% and 40% protection respectively. The peptides showed the production of Total IgG antibodies and cytokines (IL-2, IL-4, IL-6, IFN-γ, and TNF-α), indicating a possible activation of the Th1 type response. However, groups G3, G5, G6, and G8 showed production of IL-17. None of the study groups showed IL-10 production. The immunogenicity of the peptides was not enough to protect these animals and it is believed that the use of adjuvants based on PAMPs may improve the immune response offered by these peptides.

## Introduction

Caseous Lymphadenitis (CLA) is a contagious chronic disease, commonly known as "core bad " [1–4]. It is a subclinical disease that causes granuloma in the lymph nodes of the small ruminant [5]. It can be classified as superficial and visceral. The most frequent form is superficial, characterized by the development of granulomas in the superficial lymph nodes, subcutaneous tissues. However, granulomas can also be developed in internal organs, such as lungs, kidneys, liver, and spleen, showing the two forms of the disease [6]. The visceral form is characterized as asymptomatic since the current diagnosis is the presence of granulomas. CLA is a global impact disease due to its prevalence and because it causes high losses in the herd [7].

The etiologic agent of CLA is a Gram-positive bacterium, *Corynebacterium pseudotuberculosis*, present pleiomorphic forms ranging from coccus to Baston filamentous which can be between 0,5–0,6 μm for 1–3 μm size [8]. The transmission occurs mainly for the cutaneous vial, due to scars in the skin originated by management procedures that cause skin wounds such as shearing, castration, and animal markings, by aerosols or even by eating contaminated food, seem to contribute to the disease dissemination [9–11].

In this context, the diagnosis becomes paramount, as it allows the identification asymptomatic animals, which are a potential source of spread of infection in the herd, guaranteeing the execution of immediate procedures for treatment and isolation of the contaminated animal [6, 12]. These immediate procedures are also called prophylaxis, which occurs by wound asepsis and sterilization of environments contaminated by *C*. *pseudotuberculosis*, since this microorganism can remain viable in the soil for up to two years, even in dry climates and with a high incidence of light solar. Immediate procedures also allow more careful monitoring and evaluation of the herd, to avoid contact between infected and healthy animals [5, 13].

However, these measures have low efficacy due to the diagnosis of the disease being performed late (in an advanced clinical stage) and the commercial vaccines do not have high levels of protection [5, 11]. Due to the formation and development of granulomatous lesions, treatment with antibiotics becomes a procedure with low prospects, the formation of the abscess capsule almost completely prevents the diffusion of antibiotics to the region of the lesion, preventing the contact of the bacteria with the drug [14]. Some vaccines are already available on the market, but they offer important considerations about their use, as they present different levels of protection in goats and sheep [15].

Because of this, several vaccine strategies appear to supply the problems of the traditional ones. Second-generation vaccines are safer and their use is growing, among them, the use of synthetic peptides stands out, which today have become more viable due to the immunoinformatic software, which can select the dominant antigenic determinants of the protein target [16]. Droppa-Almeida, Franceschi, and Padilha (2018) [17] used bioinformatics tools to find immunodominant epitopes in endoglycosidase (CP40) which already has their immunogenic profile proven to be evaluated [18, 19], found six B cell epitopes and after evaluated the interaction with the TLR2 receptor through molecular docking, all were reactive, showing to be a potential target for vaccine composition. Thus, the main goal of the work was to evaluate the protective potential of six immunodominant epitopes obtained by immunoinformatic of the CP40 protein against Caseous Lymphadenitis in mice.

## Material and methods

### Bacterial and plasmid

The bacterial strains used in this work was the strain *E*. *coli* BL21 Star, which was cultured in Luria-Bertani (LB) or LB-Agar medium at 37°C for 18 h. When necessary, 100 μg / mL of the antibiotic ampicillin was added to the culture medium as a way to select the bacteria that had the plasmid for heterologous expression. A 1 mM solution of Isopropyl β-D-Thiogalactoside (IPTG) was used to induce the production of the protein under study. The isolated strain of *C*. *pseudotuberculosis* isolated from sheep in the Nossa Senhora da Glória city in the state of Sergipe was named CPNS and was grown in BHI broth (Brain Heart Infusion) and 5% sheep blood Agar at 37°C for 48 h. Additionally, the plasmids used were the pAE / CP40 obtained in the work of Droppa-Almeida et al. (2016) [19].

### Cloning, expression, and purification

The plasmids pAE / CP40 [19] were heat-shock transformed into *E*. *coli* BL21 Star expression strain and inoculated in LB medium containing the inducer (IPTG 1 mM), incubated on an orbital shaker at 37°C for 3 h. The culture (500 mL) was centrifuged at 4°C, 14,000 x g for 15 min, and pellet was resuspended in 40 mL wash solution (200 mM NaH2 PO4, 500 mM NaCl, 5 mM Imidazole, 8 M Urea pH 8.0) added with 100 mg / mL lysozyme and sonicated in 5 cycles (4 x 15s, at 20 kHz) and kept under stirring at 4°C for 16 h. The recombinant protein was purified by nickel affinity chromatography using column Sepharose (HisTrapTM HP—GE) using the manufacturer’s directions. Subsequently, the proteins were dialyzed in cellulose bags (25 nm x 16 nm) (Sigma) for this used 1 x saline phosphate buffer (PBS) / 0.2% Urea, with two exchanges per day for three days at 4°C, leaving proteins in a final concentration of approximately 2 mM urea. The purity of the rCP40 was determined by 12% sodium dodecyl sulfate-polyacrylamide gel electrophoresis (SDS-PAGE), and the concentration was determined using a BCA kit (Pierce).

### Epitope prediction

**Fig 1:**
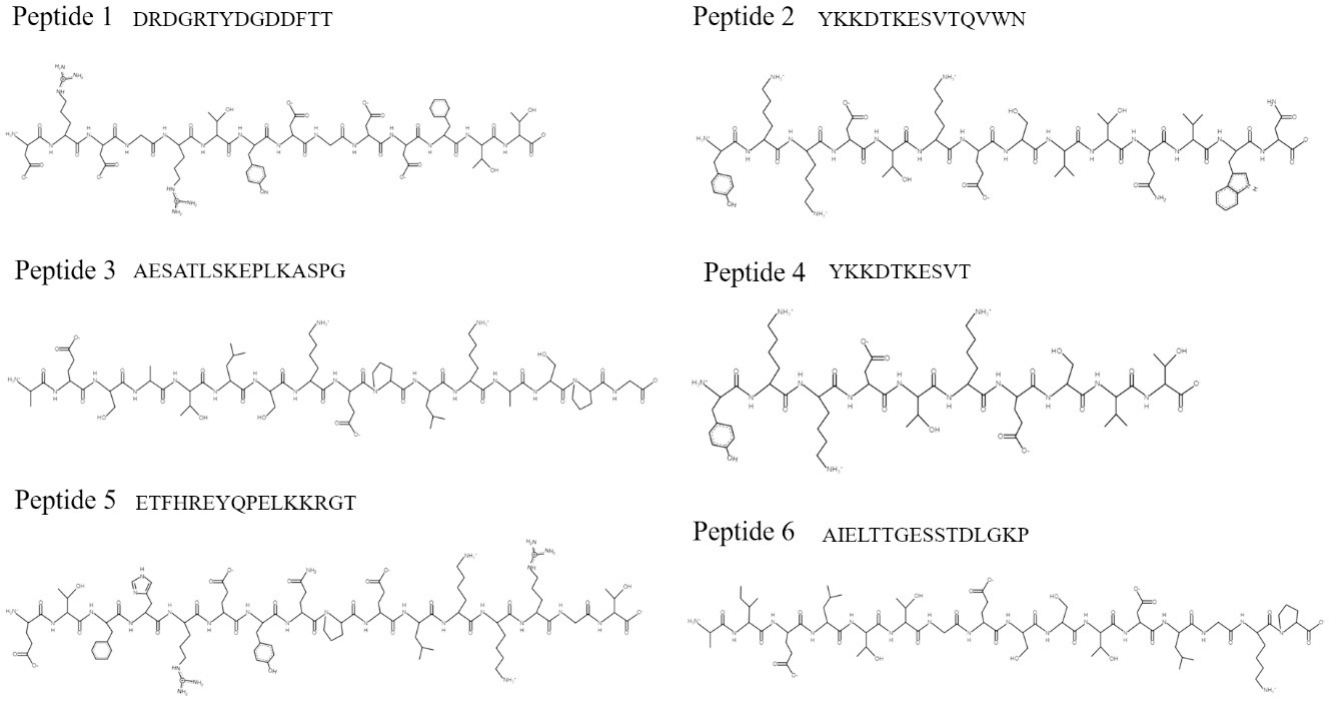
Peptides chemical structure by PepDraw.

The epitope was obtained by Droppa-Almeida, Franceschi, Padilha (2018) [17]. Previously the endoglycosidase corynebacterium (CP40) (access number in GenPept APC93958) was used to immunodominant epitope prediction of B Cell using BepiPred, ABCPred, BCPred, and Immune Epitope Database and Analysis Resource. The epitopes using in this work are available in Fig 1. The peptides after selection were synthesized by the solid-phase chemical method by AminoTech^®^ with high degree purification. The lyophilized peptides were prepared to follow manufacture instruction.

**Fig 2:**
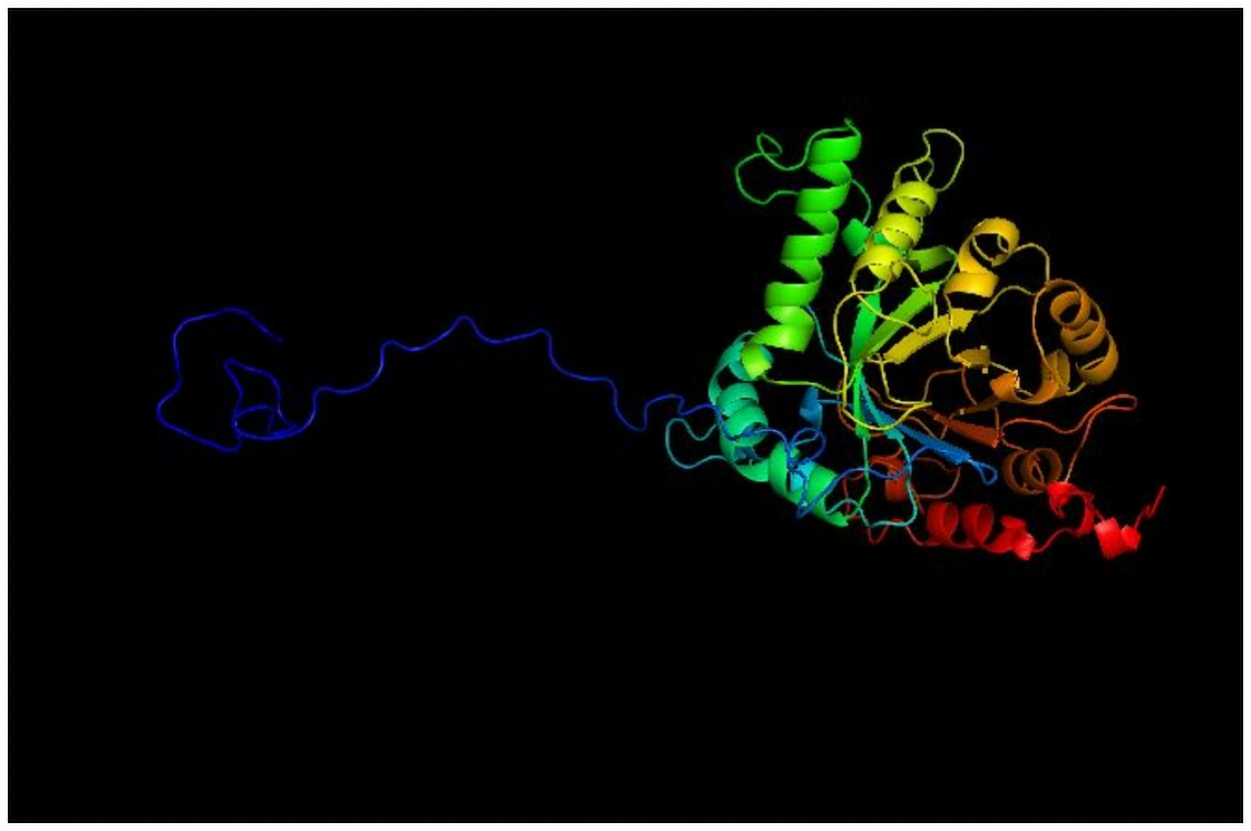
The tridimensional model predicts by Droppa-Almeida, Franceschi, and Padilha (2018) [17]. This model was used in this work to verify immunodominant epitope localization with Rasmol software.

To verify the epitope localization in CP40, the Rasmol software version 2.7 was used. The 3D model used was determined by Droppa-Almeida, Franceschi, Padilha (2018) [17] (Fig 2).

### Antigenicity evaluation by Enzyme-Linked ImmunoSorbent Assay (ELISA)

For the evaluation of the antigenicity of the positive and negative CLA negative sheep serum, they were used in an indirect ELISA assay following the methodology Oliveira, Langenegger and Meyer (1992) [20]. The immunoassay by ELISA 96-well plates, high binding was sensitized with 3μg of peptides each well in Carbonate-Bicarbonate Buffer (0,05M, pH 9,6) and incubated for 16 h at 4°C. Subsequently, the plates were washed 3 times with PBS-T (PBS 1X, pH 7.4, 0.1% tween 20) and blocked with 100 μL/well of 5% non-fat milk in PBS (1 h at 37 °C). Then, the plates were washed again, and 100 μL/well of sheep serum (1:50 in PBS-T) was added in duplicate in the plate. After 1 h of incubation at 37°C and more washes, 100 μL/well of anti-sheep IgG conjugated with horseradish peroxidase (1: 4000; Sigma Aldrich) was added (1:10.000). After five more washes, 50 μL/well of TMB (Bio-Rad) was added, and the plates were incubated for 15 min at room temperature and in the dark to reveal the results. To stop the reaction, 25 μL/well of H_2_SO_4_ solution (4N) was added. Optical density (OD) was determined by an automatic photo colorimeter for ELISA (BIO-RAD) at 495 nm.

### Ethics statement

The animal experiments in this study follow all guides by the Brazilian Society of Laboratory of Animal Sciences and by Animal Experimentation Ethics Committee (National guide follow of low 11.794 on October 8, 2008) of Tiradentes University with the number of process 010515RR approved in 2015, Jun 30.

### Peptides immunization

#### Animals

For immunization assays, female *Swiss* mice (9-12-week-old) susceptible to *C*. *pseudotuberculosis* infection were used. Animals were provided by Bioterium of Tiradentes University. They were maintained at the same place. All mice were allowed to have free access to water and maintenance diet ad libitum in a 12- hour light/dark cycle, with room temperature at 21 ± 2°C. All cages contained wood shavings, bedding, and a cardboard tube for environmental enrichment. The experiments were carried out according to the National Institute of Health, Brazil. All efforts were made to minimize suffering.

#### Immunization, challenge and blood collection

The assay was compost into nine groups with 12 animals each, 108 animals in total. Animals were divided into Control Group (G1) inoculated with saline solution, (G2) inoculated with 7,5 μg of saponin adjuvant, and G3, G4, G5, G6, G7, and G8 were inoculated with each peptide associated with 7,5 μg of saponin adjuvant. G9 group was inoculated with rCP40 associated with 7,5 μg of saponin adjuvant. The vaccines were injected by subcutaneous route with a two-week interval. Thirty days after the last immunization, *C*. *pseudotuberculosis* virulent strain CPNS (1,0 x 10^7^ CFU) was intraperitoneally injected in all mice to evaluate the protection level of immunization. During the immunization period, the animals were monitored every 3 days, but after challenge assay (60 days), animals were evaluated every day for the sites of challenge inoculation, the internal organ, lymph nodes were examined for internal abscesses and samples were collected from abscesses for, reisolation of *C*. *pseudotubercuosis* or any other clinical sign of the disease (CLA). The animals that presented morbidity, abscesses at the site of inoculation or at lymph nodes and weight loss were euthanized. Once animals reached endpoint criteria they were euthanized, preventing their suffering. By the end of 60 days, all surviving mice were euthanized by CO_2_ inhalation under anesthetic effect using IACUC approved chambers and efforts were made to minimize suffering and discomfort. For serological tests, anesthetized animals were bled from the tail vein at 0, 15, 30, 60, and 120 days after the first immunization. Sera were stored at—20°C until analysis. On the same day of the challenge, but before two mices each group was euthanized to spleen removal for cell culture and cytokine dosage.

For evaluation of protection, clinical signs of CLA were observed. The animals that died after the challenge were analyzed for the presence of granulomas, as well as the identification and confirmation of *C*. *pseudotuberculosis*. The surviving animals after the monitoring time were euthanized and the presence of granulomas or any clinical sign of CLA was analyzed.

For the analysis of vaccine efficacy after challenge, the equation used was:

$$Efficacy=\frac{CG}{Animals}-\frac{EG}{Animals}=\frac{RG}{Animals}÷\frac{CG}{Animals}=Ef\;\times 100$$

GC = control group;

EG = experimental group;

RG = result of previous subtraction;

Animals = number of animals per group;

### Immunological response evaluation

#### Humoral response

To determine the kinetics of specific antibodies (total IgG and the IgG1 and IgG2a isotypes) for the synthetic peptides of the immunized animals, indirect ELISA assays were carried out following the Oliveira et al. (1992) [20] methodology. In the sensitization stage, rCP40 was used in all analyzes.

#### Cellular response

*Splenocytes culture*. Thirty days after the last immunization with peptides vaccines, one mice of each group was euthanized and was spleen removal. The animal’s spleen was washed with RPMI 1640 supplemented (RPMI 1640 Gibco BRL add of 100 U/mL of streptomycin—LGC Bio) after by divulsion the splenocytes cells were isolated. Obtained cells were diluted at 1:100 (30 μl of acetic acid, 960 μl of trypan blue stain, and 10 μl of cells) and counted in a Neubauer chamber to determine cellular viability as well as to adjust concentration levels. Splenic cells were then cultured in 24-well plates at a final concentration of 10^6^ cells per mL. In this 24-well plate, the splenocytes were stimulated with mitogen pokeweed (Lectin of *Phytolacca americana*) (Positive control), with RPMI (Negative control) and with rCP40 in two concentrations (20 μg/mL and 40 μg/mL). The splenocytes were cultured with 5% CO_2_ at 37°C for 72 h. The culture’s supernatant was collected after 72h and maintained at -20°C.

*Cytokine dosage—flow cytometry*. The cellular response was determined by flow cytometry and was used BD Cytometric Bead Array (CBA) mouse Th1/Th2/Th17 cytokine kit, which doses IL-2, IL-4, IL-6, IFN-γ, TNF-α, IL-17, and IL-10. The equipment used was FACSCalibur—Becton Dickison and to analyze was FCAP Array Software v3.0.

### Statistical analysis

Statistical analyses were performed using GraphPad Prism version 8.0 for Windows (GraphPad Software, San Diego, CA). Data homogeneity was verified with a Kolmogorov–Smirnov test, which resulted in parametric data. Analysis of variance was performed using the One-Way ANOVA to compare sample variations in the production levels of IgG, isotypes, and cytokines, followed by Tukey’s post hoc test. A Fisher test and log-rank sum test were used to determine significant differences in mortality and survival, respectively, between the groups immunized with the peptides vaccine and the negative control groups. To verify the efficacy of the vaccine used the Epi Info^™^ software. Significant statistical differences were considered at p-values of <0.005.

## Results

### Protein purification

**Fig 3:**
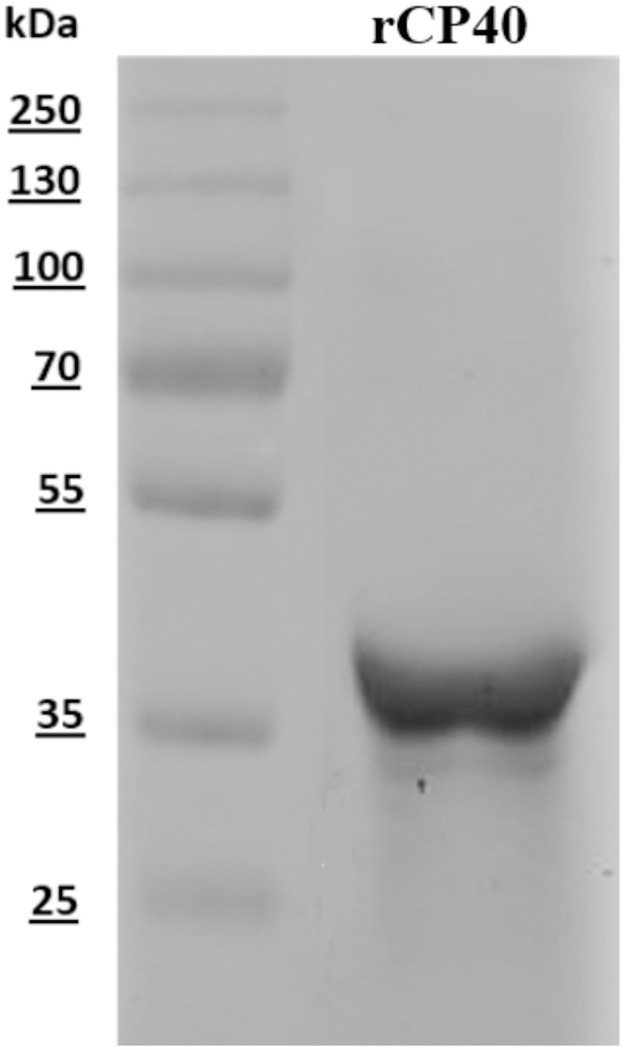
SDS-Page for purification evaluation. Purification of rCP40, literature compatible molecular weight, 40kDa.

The expression of the recombinant proteins was confirmed by SDS-Page with and the clear bands indicated the successful expression and purification of the proteins. Proteins were expressed in inclusion bodies in the *E*. *coli* BL21 Star (DE3) strain and were solubilized in 8 M urea. The yields obtained after purification were approximately 7,5 mg/L for the rCP40 proteins. The recombinant protein had the expected molecular weights 40 kDa for rCP40. Fig 3 showed rCP40 purified and the recombinant protein had the expected molecular weights by SDS-Page.

### Epitope local in CP40 protein

**Fig 4:**
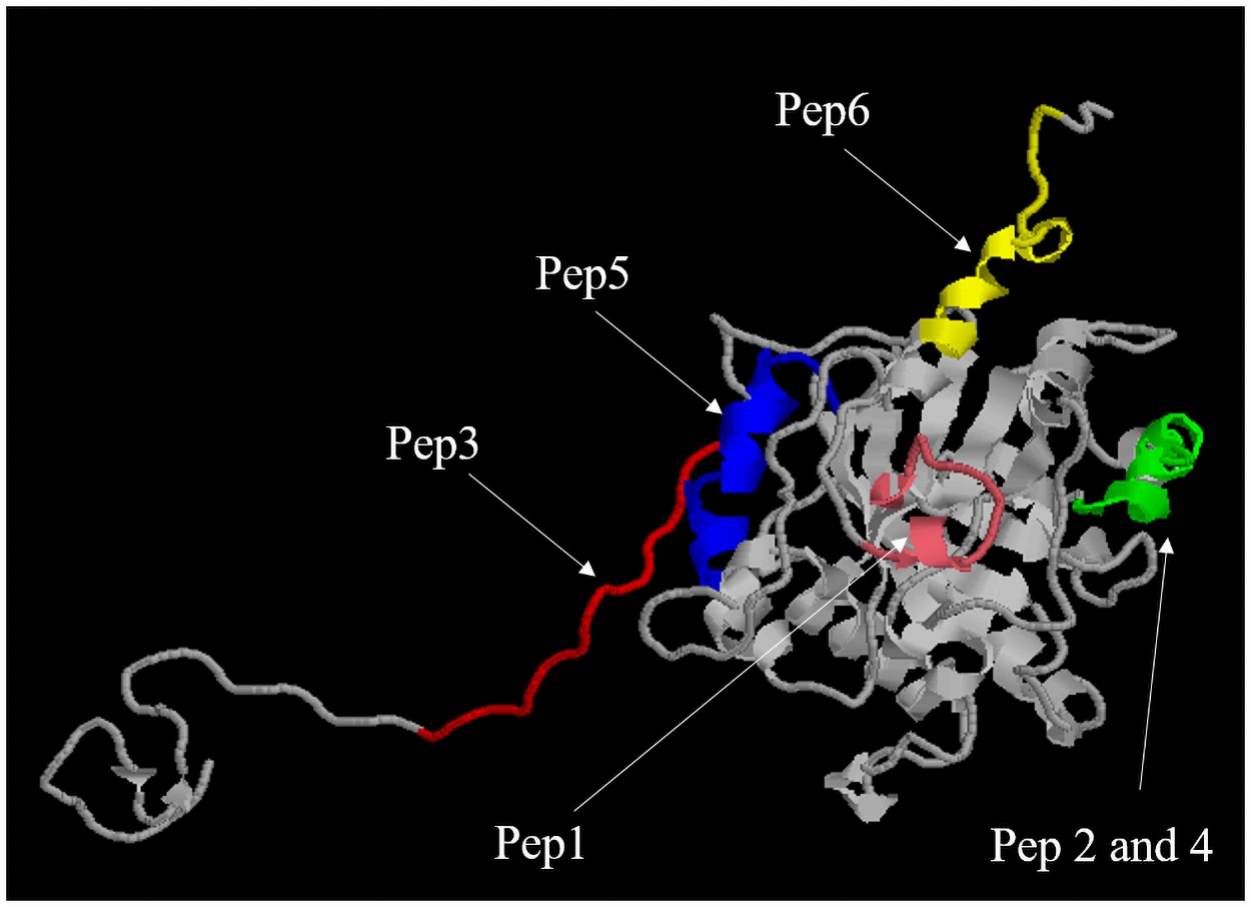
3D model of CP40 looking at the immunodominant epitope local. Peptide 1—pink; Peptide 2 and 4—green; Peptide 3—red; Peptide 5—blue and Peptide 6—yellow. This 3D model was predicted by *abnitio* for Droppa-Almeida, Franceschi and Padilha (2018) [17], and used by Rasmol to look the local for each epitope.

To verified the immunodominant epitope local in CP40 protein was used Rasmol. In Fig 4 it’s possible to see the colorful regions for each epitope.

### Antigenicity evaluation by Enzyme-Linked ImmunoSorbent Assay (ELISA)

**Fig 5:**
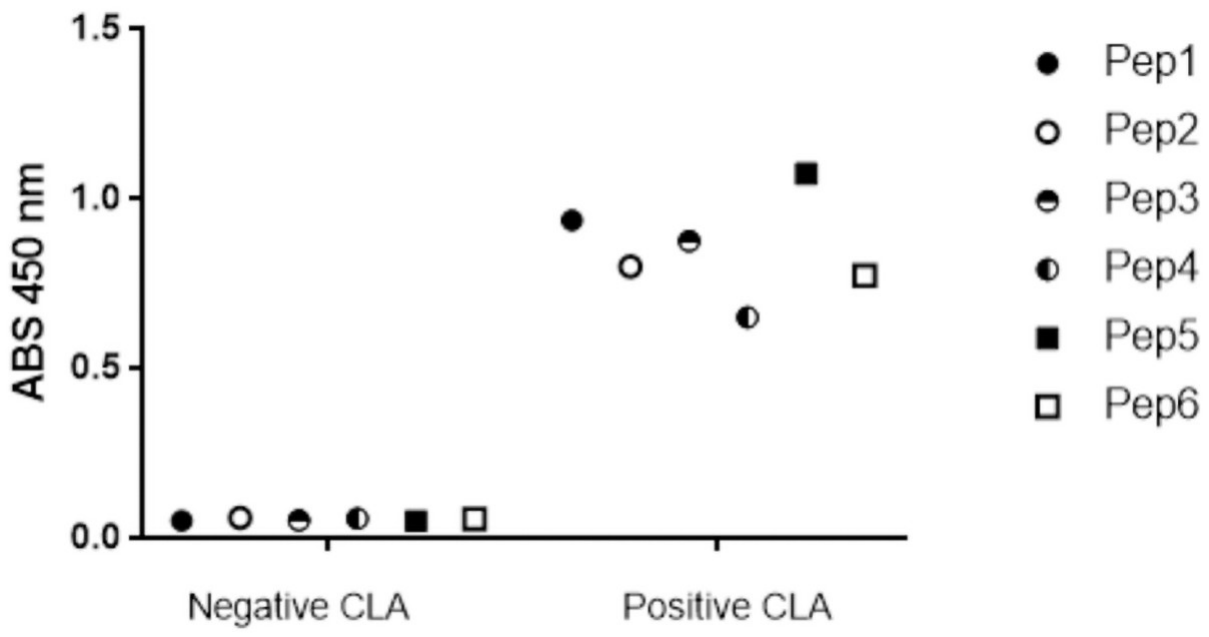
Antigenicity assay of epitopes obtained through bioinformatics tools with naturally infected animal serum (positive for CLA) and healthy serum (negative for CLA) from goat and the determined by ELISA.

To evaluate the antigenicity of synthetic peptides, positive and negative sheep sera for CLA were used. Fig 5 showed the absorbance obtained with peptides and serum reactions. This assay showed the presence of specific antibodies to CLA positive animals capable of recognizing the peptides, proving that these epitopes are linked to the formation of the humoral immune response.

### Peptides immunization

**Fig 6:**
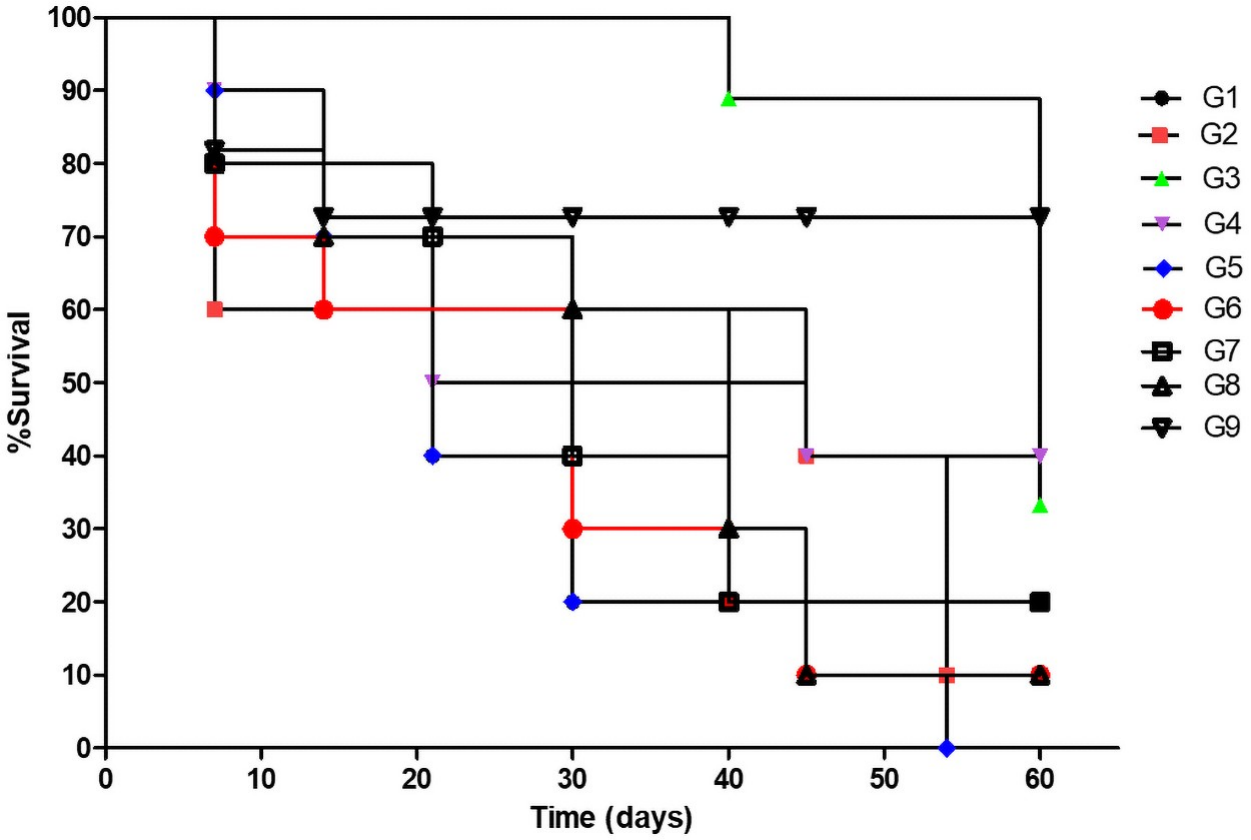
Survival curve of the animals immunized with the different vaccine formulations after challenge with the virulent strain CPNS (10^7^ CFU/mL) of *C*. *pseudotuberculosis*. Each twice-vaccinated group was composed of 12 animals and survival was monitored for 60 days after challenge. G1—inoculated with PBS; G2—inoculated with Saponin; G3—inoculated with Peptide 1 + saponin; G4—inoculated Peptide 2 + saponin and G5—inoculated with Peptide 3 + saponin; G6—inoculated with Peptide 4 + saponin; G7—inoculated with Peptide 5 + saponin; G8—inoculated with Peptide 6 + saponin and G9—inoculated with rCP40 + saponin. Statistical analysis with test Fisher P Value> 0.0076 showing no statistical difference between groups. Statistical analyses were carried out with GraphPad Prism 8.03 software systems (GraphPad Prism Software).

All peptides were used in immunization assay. For this, the assay was compost into nine groups with 12 animals each. Animals were divided into G1, G2 G3, G4, G5, G6, G7, G8 and G9. The vaccines were injected by subcutaneous route with a two-week interval. Thirty days after the last immunization, *C*. *pseudotuberculosis* virulent strain CPNS (10^7^ CFU) was intraperitoneally injected in all mice to evaluate the protection level of immunization. During challenge assay (60 days), animals were evaluated every day for the sites of challenge inoculation, the internal organ, lymph nodes were examined for internal abscesses and samples were collected from abscesses for, reisolation of *C*. *pseudotubercuosis* or any other clinical sign of the disease (CLA). Looking for the survival curve (Fig 6). The G5, G6, G7 and G8 groups did not present protection, despite showing 10% survival of animals, when compared with the control group G1 and G2 both were not able to provide protection. The controls G1 and G2 survival (10% each). The G3, G4 and G9 groups provided 12,5%, 25% and 62,5% protection against CLA, respectively. In our study, the animals of G5 died, while de control group 10% of animals survived. This can be explained by the genetic variation of the animals, since the study used *Swiss* mice, which are not isogenic. Furthermore, it should be noted that even with 10% survival in the G1 and G2 controls, there was no statistically significant difference for G5, which showed 100% death. The fact of having survival in some animals in the control group has already been demonstrated in other studies with vaccines for CLA as in the study of Pinho et al. (2021) [21] where the control group also had 10% survival.

Through statistical analyzes by Fisher’s test, there were no statistical differences between the control and experimental groups.

All dead animals were analyzed for the presence of clinical signs of CLA. When comparing in relation to clinical signs, the control groups showed a greater number of granulomas than the experimental groups. The presence of granulomas was found in all animals killed after challenge. These granulomas were removed, sown on BHI agar and after isolation the biochemical analysis was carried out confirming the presence of *C*. *pseudotuberculosis*. The surviving animals after the monitoring time were euthanized and no granuloma or other clinical signs of CLA were observed. In view of these results, it appears that the animals that survived in the experimental groups were protected by the vaccine and even those that died from CLA had an inferred number of granulomas to compare with the control groups G1 and G2.

### Humoral response

**Fig 7:**
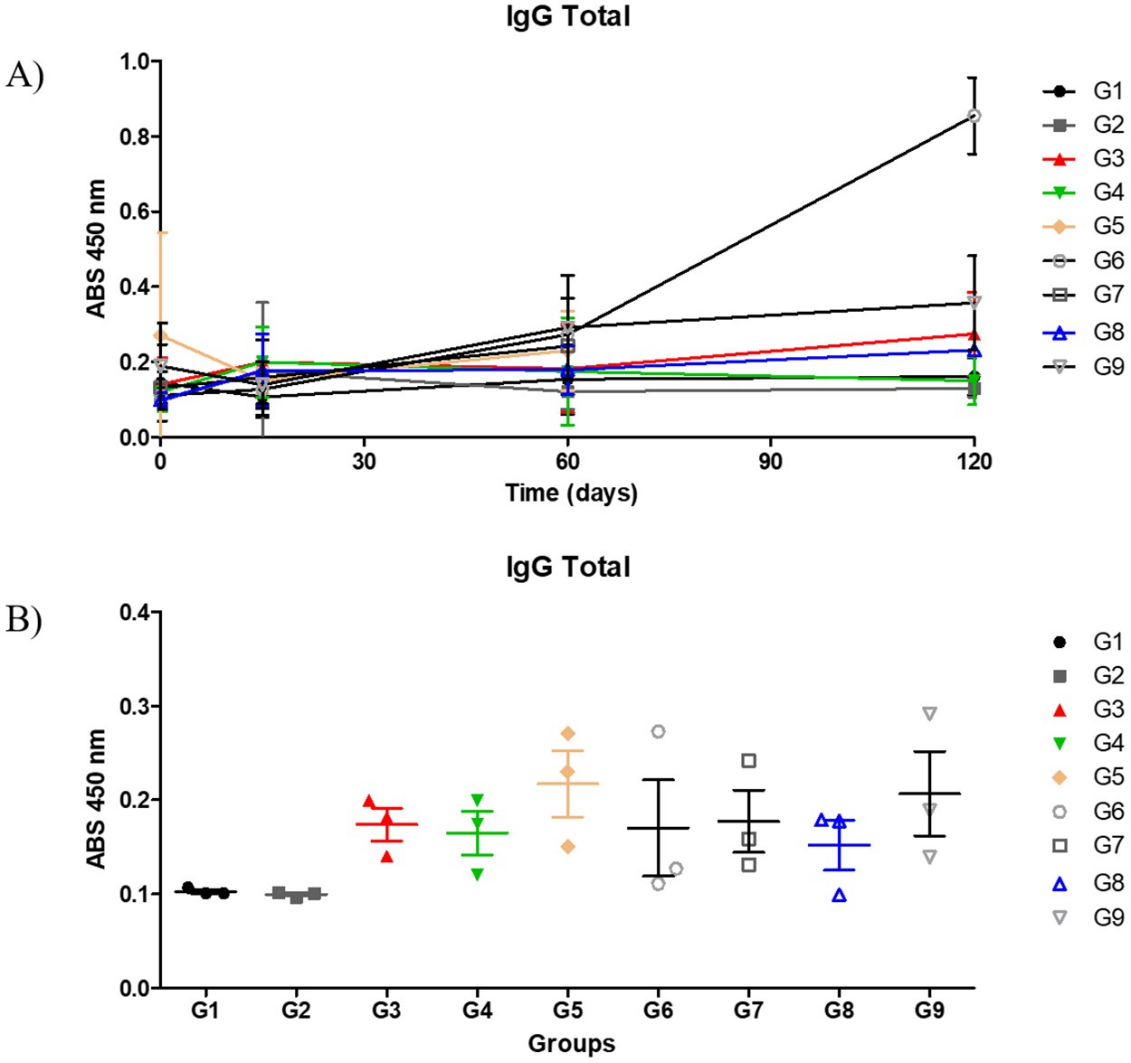
IgG Total antibody levels in mice immunized with different peptides. The results are presented as the mean and standard deviation (bars) of absorbance (nm) in the indirect ELISA assay for each experimental group with 12 animals. A) G1—inoculated with PBS; G2—inoculated with Saponin; G3—inoculated with Peptide 1 + saponin; G4—inoculated Peptide 2 + saponin and G5—inoculated with Peptide 3 + saponin; G6—inoculated with Peptide 4 + saponin; G7—inoculated with Peptide 5 + saponin; G8—inoculated with Peptide 6 + saponin and G9—inoculated with rCP40 + saponin. The blood was collected and evaluated on day 0 (pre-immune) and day 15 (before the second immunization) and 60 after the first immunization. Each ELISA was performed with rCP40. B) Immunogenicity of peptides and experimental groups on days 0, 15 and 60.

To verify the humoral response with peptides ELISA was performed for the determination of total IgG (Fig 7). Regarding the humoral response, Fig 7A showed the kinetics of total IgG antibodies throughout the experiment. In relation to the groups immunized with the peptides, a greater number of specific antibodies is perceived in the first 15 days for the G4, G5 and G8 group and after the challenge the G6 group showed an increase in the production of these antibodies and this may be due to the presence of the bacteria, since in relation to the survival curve and the presence of granulomas this group had low protection.

To try to better understand, Fig 7B showed the first 60 days of vaccination, referring to the immunogenicity of the peptides in relation to the control groups G1, G2 and G9 (inoculated with rCP40). It is possible to observe the immunogenicity of synthetic peptides before the challenge, and in this context, the greatest absorbencies are observed by groups G5, G6 and G7 on day 60. While the groups G3, G4 and G8 showed similar absorbencies in relation to the presence of IgG total values for each peptide evaluated.

When analyzing the results of the reactive antibodies against all groups the total IgG production presented in the statistical differences P value> 0.005.

**Fig 8:**
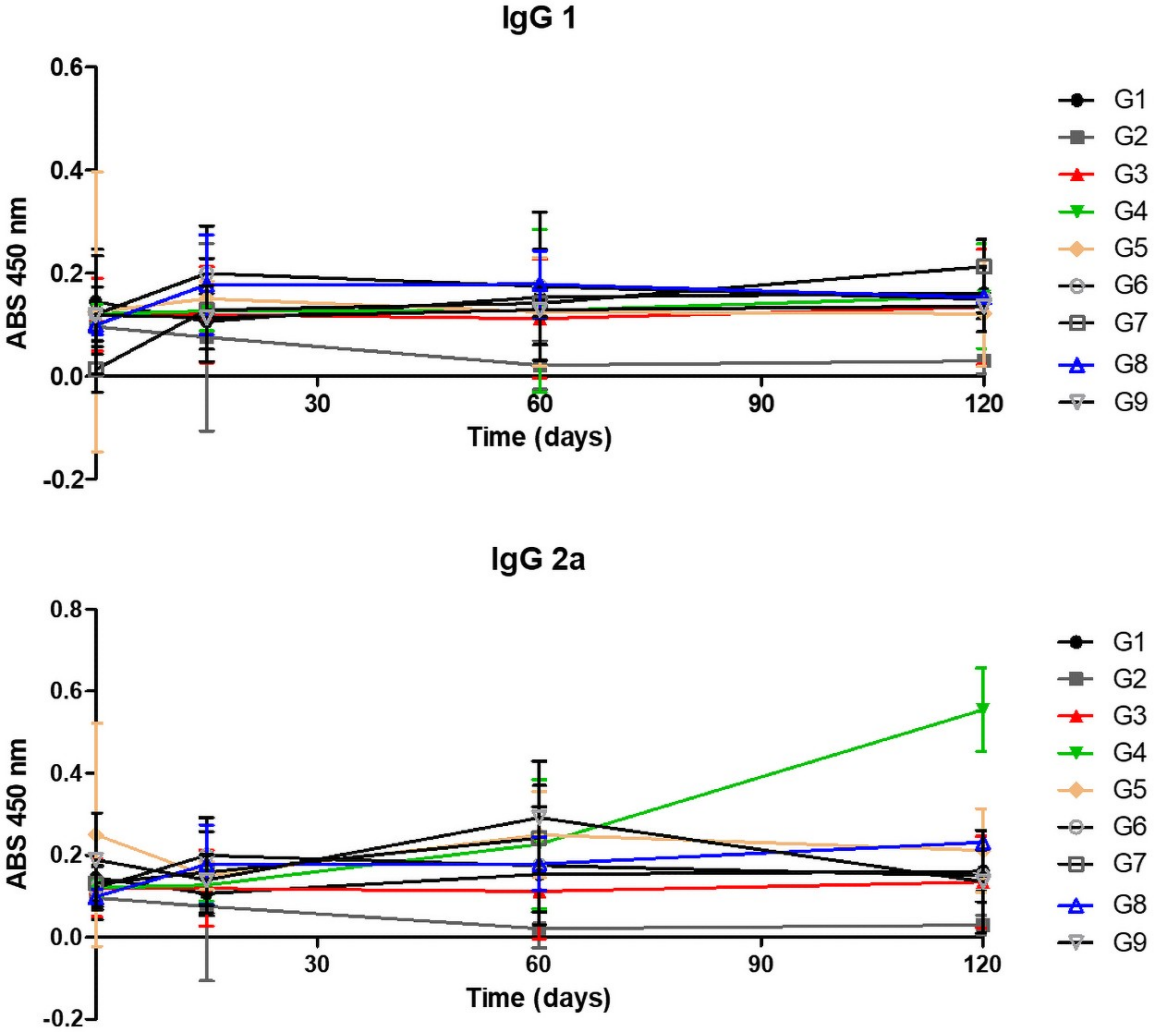
IgG1 and IgG2a antibodies levels in mice immunized with different peptides. The results are presented as the mean and standard deviation (bars) of absorbance (nm) in the indirect ELISA assay for each experimental group with 12 animals. G1—inoculated with PBS; G2—inoculated with Saponin; G3—inoculated with Peptide 1 + saponin; G4—inoculated Peptide 2 + saponin and G5—inoculated with Peptide 3 + saponin; G6—inoculated with Peptide 4 + saponin; G7—inoculated with Peptide 5 + saponin; G8—inoculated with Peptide 6 + saponin and G9—inoculated with rCP40 + saponin. The blood was collected and evaluated on day 0 (pre-immune) and day 15 (before the second immunization) and 60 after the first immunization. Each ELISA was performed with rCP40.

To try to understand the immunologic response type acquired in immunizations, the IgG isotypes were analyzed: IgG1 and IgG2a (Fig 8). It’s possible to realize that G4 was the group with high levels from IgG2a. The results of IgG1 isotype didn’t show high levels and all groups showed the same reactivity. Both kinetics didn’t show a statistical difference, the P-value was 0.1248 and 0.2473 to IgG2a and IgG1 respectively. Given the diversity of Ig molecules, the IgG1, IgG2a, IgG2b and IgG3 subclasses were selected due to the capacity that these subclasses have in helping to understand the profile of the immune response. As is already known, the ideal immune response to protect animals against CLA is of the Th1 type, which leads to the production of IgG2a and IgG2b subclasses. While the Th2 profile promotes an increase in the production of IgG1 and IgG3 subclasses in mice.

### Cellular response

**Fig 9:**
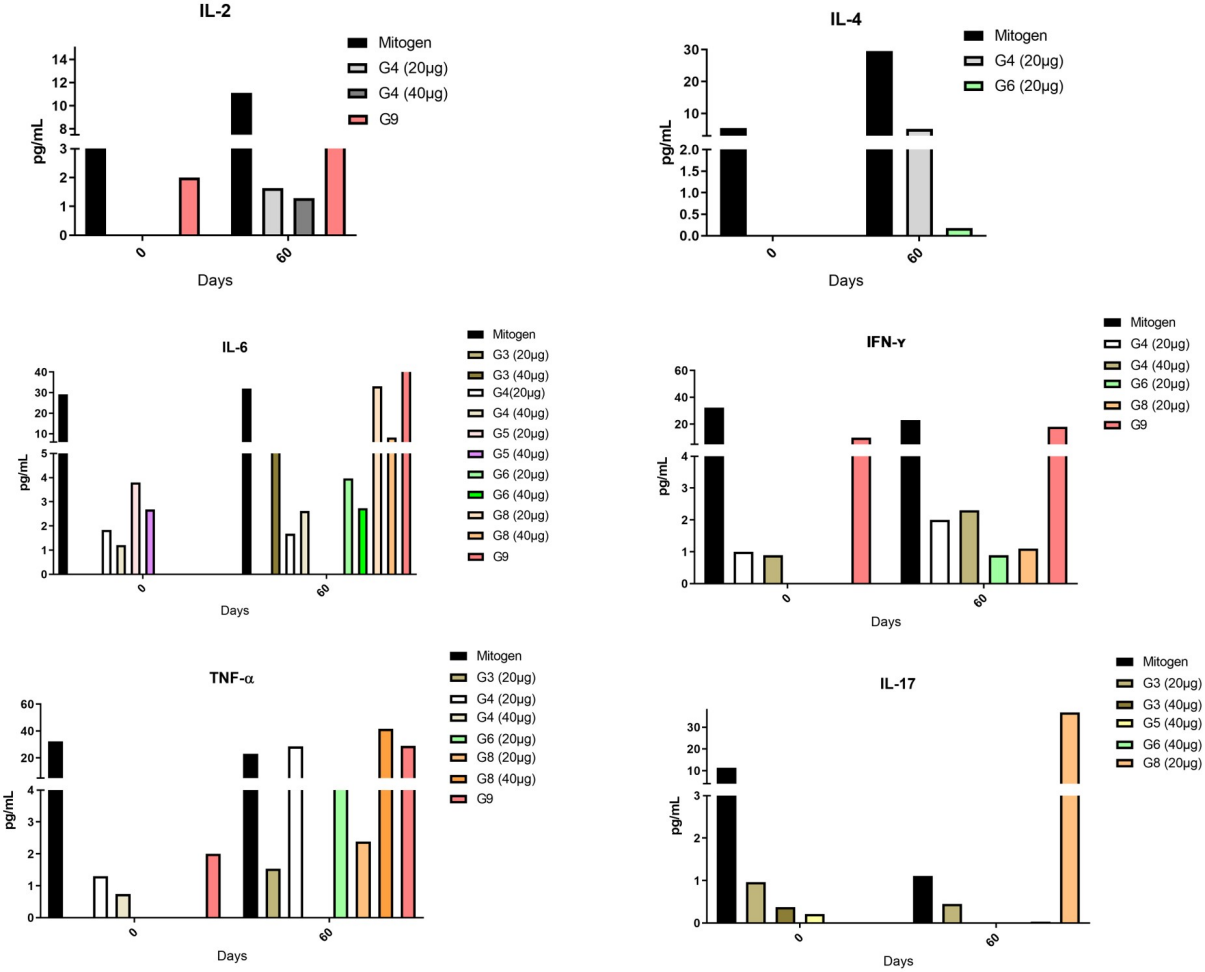
Cytokines profile of mice immunized with different peptides and their control. Thirty days after the last immunization with peptides vaccines, one mouse each group was euthanized and were spleen removal. The splenocytes were isolated and assayed for the production of IL-2, IL-4, IL-6, IFN-γ, TNF-α, IL-17, and IL-10 by flow cytometry and were used BD Cytometric Bead Array (CBA) mouse Th1/Th2/Th17 cytokine kit. The results are presented as mean ± standard error and were statistically evaluated by one-way ANOVA and Tukey’s post-test. G1—inoculated with PBS; G2—inoculated with Saponin; G3—inoculated with Peptide 1 + saponin; G4—inoculated Peptide 2 + saponin and G5—inoculated with Peptide 3 + saponin; G6—inoculated with Peptide 4 + saponin; G7—inoculated with Peptide 5 + saponin and G8—inoculated with Peptide 6 + saponin; G9—inoculated with rCP40 + saponin. The splenocytes were stimulated with mitogen pokeweed (Lectin of *Phytolacca americana*) (Positive control), with RPMI (Negative control) and with rCP40 in two concentrations (20 μg/mL and 40 μg/mL).

To try to understand the profile of the immunological response, the cytokines IL-2, IL-4, IL-6, IFN-ɣ, TNF-α, IL-17, and IL-10 were measured by flow cytometry. The result of the cytokines determination was low when compared with the mitogen that was used as a positive control, for the assay and IL-10 were not present in any evaluated group. In Fig 9 it is possible to verify the peptides that were shown to be reactive for each cytokine specifically. The results on IL-2 only showed production by the G4 at point 60 after the challenge in the surviving animals, it was present in both tested concentrations (20μg and 40μg). Regarding IL-4, it is possible to verify the production by the groups G4 and G6 both after the challenge, unlike IL-6 which was absent in the animals inoculated with G7, the other groups showed production of this cytokine. Regarding the production of IFN-ɣ only groups inoculated with G4, G6 and G8 presented the production of this cytokine. The production of TNF-α was present in the groups G3, G4, G6, and G8. In groups G3, G5, G6, and G8, IL-17 was present. G9 was the group inoculated with rCP40 and showed the production of cytokines IL-2, IL-6, IFN-γ, and TNF-α. IL-2, IFN-γ, and TNF-α were present before the challenge, while the presence of IL-6 was present after the challenge, and this is due to the presence of *C*. *pseudotuberculosis*. As in previous work, rCP40 tends to direct a th1 type response, corroborating the expressions of cytokines present before the challenge. kinetics didn’t show a statistical difference, the P-value was 0.2348.

## Discussion

Immunodominant epitopes are specific regions within a protein recognized by immunologic receptors, widely used for vaccine composition. The epitope database is a collection of viruses, bacterium, fungi, and protozoa epitope. However, the search needs to be directed to the type of immunological response to be activated, the effective response against *C*. *pseudotuberculosis* is a Th1 response [11], therefore, the search for MHC class II epitopes for B cells is important.

The CP40, characterized by an endoglycosidase, is an important virulence factor of *C*. *pseudotuberculosis* and the immunogenic activity was tested [18, 19, 22]. For this, this protein was tested *in silico* for immunodominant epitope predicted by Droppa-Almeida, Franceschi and Padilha (2018) [17], the epitope selection was directed for 10–16 amino acids residues size, the ideal size to bind into the slit of the MHC II molecule. The choice of prediction for MHC II epitopes is due to the targeting of the immune response, which will lead to the activation of TCD4 + lymphocytes, important for the activation of T and B lymphocytes [23].

After that, the peptides were tested by ELISA with naturally infected serum and heathy serum from goat to antigenicity evaluation. All peptides presented interaction with positive serum, showing that all epitopes are linked to the formation of the humoral immune response. These animals were exposed to natural infection and due to frequent CP40 presence with virulence factors, there are memory antibodies in serum. This is an important result showing a specificity and confirmability bioinformatic tools have in epitope prediction, which would only be possible with obtaining the three-dimensional structure obtained by crystallography or nuclear magnetic resonance, techniques that besides the high cost also present several limitations about the determination of the three-dimensional structures of proteins and can be predicted through the phage display technique, which demands time and high cost when compared to the use of *in silico* predictions.

These results about the survival curve reveal that synthetic peptides failed to protect animals and this is due to several factors. One of the factors was the use of the subcutaneous route, which has been widely used due to its advantages when compared with the intravenous or intramuscular route [24]. However, variable and incomplete bioavailability is a major concern since the mechanisms of subcutaneous absorption of protein therapy are not entirely understood and require further investigation [24]. The anatomy of the subcutaneous space (SC) has been widely debated regarding its immunological potential. It can be argued that, since the SC layer contains few dendritic cells, their main function is to present potent antigens to T cells and initiate the immune response [25, 26]. However, studies using the fluorescence-labeled antigen-MHC peptide complex along with CD40 screening have revealed the role of cutaneous dendritic cells in obtaining an immune response; specifically, Langerhans cells, dendritic cells resident in the epidermis and dermis [26].

There are also reports of possible depuration of this antigen at the injection site by immune cells. These antigen-presenting cells migrate to the SC space after the injection, process the administered protein, and present it to the T cells present in the lymphatic vessels as a “first pass” immune interaction before reaching the systemic circulation [27]. Thus, the migratory potential of these cutaneous dendritic cells drives the immunogenicity of proteins administered by SC [26, 27]. Another justification may be the activation of an immune response of type Th2. This type of response is not ideal for facultative intracellular pathogens allowing microbial evasion, aggravating the animal condition [28].

When analyzing a humoral response, it was possible to see that the peptides in this study were immunogenic manufactured according to each group. The G4 group, which among the groups inoculated with peptide was the one with the lowest animal mortality, with high levels of IgG2a, in this case, is important because it suggests a Th1 immune response. In mice, IgG2a production reveals a Th1 immune response, an ideal response against *C*. *pseudotuberculosis*. To resist infections by intracellular pathogens such as CLA, immune responses of the Th1 type dominated by the production of cytokines of the type IL-2, IFN-γ, TNF-α, as well as production of antibodies of the IgG2a subtype, are required, while protection from infections by extracellular pathogens is often associated with humoral immune responses dominated by the production of IgG1, IL-4, and IL-10 [29].

In this study the rCP40 group (G9) was used only for comparison, had a protection rate (70%) different from the values previously found when the rCP40 was used presenting 100% protection [19]. The rCP40 (an endoglycosidase from *C*. *pseudotuberculosis*) based vaccine has been demonstrated to mediate a sharp production of IgG2a, indicating a type 1 T-helper (Th1) cell response [19]. The use of the *Swiss* mouse was possible due to the pilot previously done to determine the LD_50_, which indicated the susceptibility to CLA, as well as the presence of granulomas, clinical signs compatible with the disease studied. In order to verify the parasite-host relationship during infection with wild type and attenuated strain of *C*. *pseudotuberculosis* [30], used the Balb/c, C57Black / 6 (wild, knockout (KO) for nitric oxide (ON), KO-IFN-γ, KO-IL-10) and *Swiss* were used, and it was found that all are susceptible to CLA in different degrees, the mice of the *Swiss* strain were consider sensitive for CLA, similar results with the isogenic line Balb /c.

In this study, we investigated IL-2, IFN-γ, and TNF-α (Th1 type cytokines), IL-4 and IL-6 (Th2 type cytokines), and IL-17 (Th17 type cytokines) expressed in splenocytes of mice, and IgG Total for a humoral response, as well as isotypes IgG1 and IgG2a. When analyzing the production of cytokines by group, initially the group G3 showed production of TNF-α and IL-6, this cytokine stimulates the production of acute-phase proteins by hepatocytes and stimulates the production of neutrophils [31–34], as well as being associated with a decrease in the action of the cellular response [35]. In turn, TNF-α cytokines act by increasing vascular permeability allowing the passage of cells recruited from the innate immune response, in addition to promoting the activation of these cells. TNF-α can also act as a coagulation inducer, being an important agent in the formation of granuloma. In this context, we can understand why the group inoculated with G3 was not able to provide adequate protection since the cytokines that were produced are not able to provide adequate protection against CLA.

When analyzing the G4 group, we verified the presence of the three cytokines aimed at a Th1 profile response, ideal against CLA, but the presence of IL-4 and IL-6 is also noted, the presence of IL-4 occurred 60 days after the challenge, when compared with the total IgG kinetics, we can relate the presence of IL-4 being responsible for the increase in total IgG, since in previous studies the presence of IL-4 was associated with the activation of B cells and IgG production [36]. The presence of IL-6 is due to the pathophysiological profile of CLA, which leads to a greater production of IL-6 which is responsible for attracting macrophages and neutrophils to the site of infection. In the G5 group, only the cytokines IL-6 and IL-17 were present from point 0, not related to infection but to the activation of the antigen itself, this group presented 100% animal mortality.

The G6 showed the production of IFN-ɣ, TNF-α, IL-17, IL-4, and IL-6 every 60 days after the challenge with the virulent strain, being more related to the pathogenesis of CLA, which presents a profile of expressed cytokines, where the pro-inflammatory cytokines TNF-α and IFN-ɣ prevail mainly at the infection site [37]. Pépin et al. (1997) [38] demonstrated in a study that the stimulation of IFN-ɣ as well as the formation of granulomas are independent of the presence of phospholipase D, considered the main virulence factor of *C*. *pseudotuberculosis*. It was also found that the expression of inflammatory cytokines such as IL-1β and TNF-α was elevated at the inoculation site, while the expression of cytokines associated with T cells, such as IL-2 and IL-4, were higher in the draining lymph node. Taking into account the levels of cytokine expression Pépin et al. (1997) [38] suggested that the formation of granuloma may be directly related to the two types of responses Th1 and Th2. Furthermore, inflammatory cytokines, which present high levels at the beginning of granuloma formation, such as IFN-ɣ, IL-2, IL-4, MCP-1, and TNF-α, are important factors in reducing the spread of the bacteria [39]. The G7 did not show cytokine production, however, the G8 showed production of IFN-ɣ, TNF-α, IL-6, and IL-17, all present only 60 days after the challenge, which may be related to the pathogenesis of CLA, as described for the G6. The G9 showed production of IL-2, IL-6, IFN-ɣ and TNF-α IL-2, IFN and TNF were present before the challenge, indicating the immunogenicity and its targeting of the immune response, while IL-6 was only present after the challenge, present only 60 days after the challenge, which may be related to the pathogenesis of CLA, as described for the G6 and G8.

Synthetic peptide-based vaccines are an alternative solution to overcome the disadvantages associated with classic vaccines. Peptide-based vaccines are constructed with protein antigens, designed to induce immune response. However, these peptides are poorly immunogenic and need to be administered with additional immunological stimulating agents such as adjuvants or particle systems/transporters. In this work, saponin was used as a vaccine adjuvant, which is based on purified fractions such as Quil A and its derivatives of QS-21, isolated from the bark of *Quillaja Saponaria Molina*, which was evaluated in several clinical trials [40]. Their unique ability to stimulate both the Th1 type response and the production of cytotoxic T lymphocytes (CTL) against exogenous antigens makes them ideal for use in subunit vaccines and vaccines directed against intracellular pathogens, as well as for therapeutic cancer vaccines [40]. For CLA two vaccines were tested with the association of saponin and presented 90–100% protection of animals [19, 41]. However, in this work, the use of this adjuvant may have contributed to the low protection of immunized animals, since this adjuvant is not based on Molecular Patterns Associated with Pathogens (PAMPs), a type of adjuvant that would be ideal for peptides, due to its low immunogenicity [42–44]. The use of PAMP-based adjuvants is increasing because it mimics an immune response, having links with some receptors in the Toll-Like family (TLR) [45, 46].

Therefore, it is noted that the studied peptides were able to activate the humoral response and distinct cellular responses, however, low production of the cytokines described above was seen. For the activation of the immune response to occur, the antigen needs to be able to stimulate a whole arsenal of receptors and cytokines that will activate the dendritic cells which will activate the virgin T cells, in vaccines based on isolated antigens, such as the use of peptides, adjuvants are needed, which are essential to enhance this activation. The use of saponin in the veterinary field is large, but when it comes to synthetic peptides based on immunodominant epitopes, it is noted that the need for more sophisticated adjuvants such as CpG-ODN, IL-12 or the glucan family itself can help better in potentiation of the immune response.

## Conclusion

Caseous lymphadenitis is a disease that causes several economic losses due to its high rate of dissemination. Thus, the best cost-benefit is prophylaxis, a strategy currently absent. The 40 kDa protein that has already been evaluated for its immunogenicity [18, 19]. The use of its polypeptide sequence was a template for obtaining six peptides between 10–16 amino acid residues, to generate peptide vaccines. Thus, after the tests, it was noticed that the peptides designed were antigenic in the serum of naturally infected animals and they were associated with the adjuvant saponin and inoculated in mice. The animals vaccinated with the peptides were not protected, however, they were able to activate the production of IgG type antibodies, with greater predominance in the production of IgG2a by G2, as well as they were also able to activate the production of cytokines such as IL-2, IL- 4, IL-6, IFN-γ, and TNF-α indicating a direction for Th1 type immune response, effective for infection with *C*. *pseudotuberculosis*. Despite the immunogenicity of the peptides tested in this work, they were not able to protect the animals and it is believed that the use of adjuvants based on PAMPs may improve the immune response offered by these peptides. It is interesting that in the future, research may be able to evaluate different adjuvants with these immunogenic peptides, because of the need to create a vaccine against CLA.
